# Visual Collaboration Leader-Follower UAV-Formation for Indoor Exploration

**DOI:** 10.3389/frobt.2021.777535

**Published:** 2022-01-04

**Authors:** Nikolaos Evangeliou, Dimitris Chaikalis, Athanasios Tsoukalas, Anthony Tzes

**Affiliations:** ^1^ Robotics and Intelligent Systems Control (RISC) Lab, Electrical and Computer Engineering Department, New York University Abu Dhabi, Abu Dhabi, United Arab Emirates; ^2^ Electrical and Computer Engineering Department, New York University, Brooklyn, NY, United States

**Keywords:** UAV, drone, swarm, leader follower, relative localization

## Abstract

UAVs operating in a leader-follower formation demand the knowledge of the relative pose between the collaborating members. This necessitates the RF-communication of this information which increases the communication latency and can easily result in lost data packets. In this work, rather than relying on this autopilot data exchange, a visual scheme using passive markers is presented. Each formation-member carries passive markers in a RhOct configuration. These markers are visually detected and the relative pose of the members is on-board determined, thus eliminating the need for RF-communication. A reference path is then evaluated for each follower that tracks the leader and maintains a constant distance between the formation-members. Experimental studies show a mean position detection error (5 × 5 × 10cm) or less than 0.0031% of the available workspace [0.5 up to 5m, 50.43° × 38.75° Field of View (FoV)]. The efficiency of the suggested scheme against varying delays are examined in these studies, where it is shown that a delay up to 1.25s can be tolerated for the follower to track the leader as long as the latter one remains within its FoV.

## 1 Introduction

The use of Unmanned Aerial Vehicles (UAVs) towards autonomous task completion has received increased attention in the past decade. Indicative tasks include aerial manipulation in Gassner et al. (2017), Gkountas and Tzes (2021), Nguyen and Alexis (2021), Li et al. (2021), surveillance in Bisio et al. (2021), Tsoukalas et al. (2021), as well as Simultaneous Localization and Mapping (SLAM) in Papachristos et al. (2019a), Tsoukalas et al. (2020), Dang et al. (2019) or inspection in Papachristos et al. (2016), Steich et al. (2016), Bircher et al. (2015); these works necessitate the collaborating agents to exchange their relative pose (position and orientation).

High frequency measurements from the inherent Inertial Measurement Unit (IMU) within each UAV are filtered using an Extended-Kalman-Filter (EKF) for attitude estimation (Abeywardena and Munasinghe, 2010) in Flight Control Units (FCUs). Migrating from the attitude to the altitude estimation necessitates the use of additional onboard sensors. Among these, the Global Positioning System GPS/GNSS (Qingbo et al., 2012) typically feeds at 5Hz positioning data to the FCU. In GPS-denied indoor environments, other sensing modalities are deployed for estimating the drone position within a swarm. These include LiDaR sensing (Kumar et al., 2017; Tsiourva and Papachristos, 2020; Yang et al., 2021), RSSI measurements (Yokoyama et al., 2014; Xu et al., 2017; Shin et al., 2020), RF-based sensing (Zhang et al., 2019; Shule et al., 2020; Cossette et al., 2021) and visual methods (Lu et al., 2018).

### 1.1 Visual Relative Pose Estimation Sensors and Techniques

Optical flow sensors in Wang et al. (2020), Chuang et al. (2019), Mondragon et al. (2010), monocular SLAM in Tsoukalas et al. (2020), Dang et al. (2019), Papachristos et al. (2019b), Schmuck and Chli (2017), Trujillo et al. (2020), Mur-Artal et al. (2015), Dufek and Murphy (2019), McConville et al. (2020) and binocular SLAM in Mur-Artal and Tardós (2017); Smolyanskiy and Gonzalez Franco (2017) are typically used to infer the pose of mobile aerial agents. These methods usually require structural features and can result in error accumulation. More recently, deep learning techniques for relative pose estimation have been introduced for accurate results (Mahendran et al., 2017; Radwan et al., 2018; Patel et al., 2021).

Regarding techniques using an external reference shape for pose extraction, these typically include passive markers, including ArUco (Xavier et al., 2017) and April tags (Wang and Olson, 2016) fiducial markers. These methods introduce a variety of an a-priori known planar, rectangular, black and white pattern, which, when in line of sight, allows the computation of its relative pose to the camera. The extension of this is relative pose estimation for every unit bearing such a marker arrangement.

### 1.2 Limitations of Pose Estimation Systems

The onboard IMU on a UAV provides both orientation and position information when coupled to a GPS/GNSS receiver. Its average positioning accuracy (Upadhyay et al., 2021) is close to 3m, with maximum errors reported close to 10m. The causes of such error are: weather conditions and signal refractions from obstacles such as large buildings in the proximity, and poor signal under bad environmental conditions (clouds, dust) (van Diggelen and Enge, 2015). Even with the use of a Real Time Kinematic (RTK) component, GPS-RTK measurements drift as much as 6.5cm in the horizontal plane and 30cm in the vertical axis under the assumption of 1h tuning of the base station prior to the measurement collection (Sun et al., 2017). Similarly, the orientation accuracy suffers from constant drifting with time (LaValle et al., 2014; Upadhyay et al., 2019) Even with the utilization of bias correction methods, such as the use of magnetometer sensors for correcting the yaw drift, an RMS error of more than 5° is found in orientation on every axis.

Onboard LiDaR sensors reduce the quantization measurement error from 0.15m up to 1m (Chen et al., 2018), depending on the number of instantaneous scans performed at each position. The main concern with respect to LiDaR is the weight (close to 1Kg) and the high acquisition cost. RSSI and RF-based methods can be a viable alternative having a positioning noise deviation of 0.1m (Cossette et al., 2021). The drawback stems with the requirement for precise placement of at least four external anchor nodes, with additional nodes required for multi-agent experimentation, and need of absence of metallic objects proximity to the antennas (Suwatthikul et al., 2017).

Another viable solution discussed is the use of onboard visual modalities for self-localization in monocular or binocular pose estimation applications. In the case of image feature extraction used in SLAM, a few centimeters error is reported (Daniele and Emanuele, 2021). However, the feature identification is computationally intensive and passive markers can be employed to ease this load. The black border of these markers assists in their fast detection, and distance error of 15cm, when placed up to 2.75m from the imaging modality (López-Cerón and Canas, 2016) have been reported.

### 1.3 Contributions

In this work the utilization of multiple markers placed on an Archimedean solid configuration is used for visual relative localization. The reported distance error is approximately 7.5cm in a hovering scenario of a dual UAV multi-agent system. The overall pose estimation duration is close to 30msec and this scheme provides robustness under varying lighting conditions and partial occlusion, despite any relative yaw-angle between these UAVs. The fiducial-marker carrying UAVs are then used in a leader/follower formation. The UAVs’ dynamical equations are linearized and the maximum allowable delay that the advocated controller can tolerate is provided. It is shown that as long as the leader remains within the follower’s Fov, the controller can tolerate more than 57 samples (missing frames). We should note that the major advantage stems from the lack of using RF-communication for exchanging the UAVs’ pose and possibly saturating the communication channel in such a case.

This article is structured as follows. The concept behind the visual localization is presented in Section 2, followed by the linearized UAV-dynamics in Section 3. The adopted controller and the maximum allowable delay in computing the relative pose is described in Section 4. Experimental studies appear in Section 5 followed by concluding remarks.

## 2 Visual-Assisted Relative Pose Estimation

**FIGURE 1:**
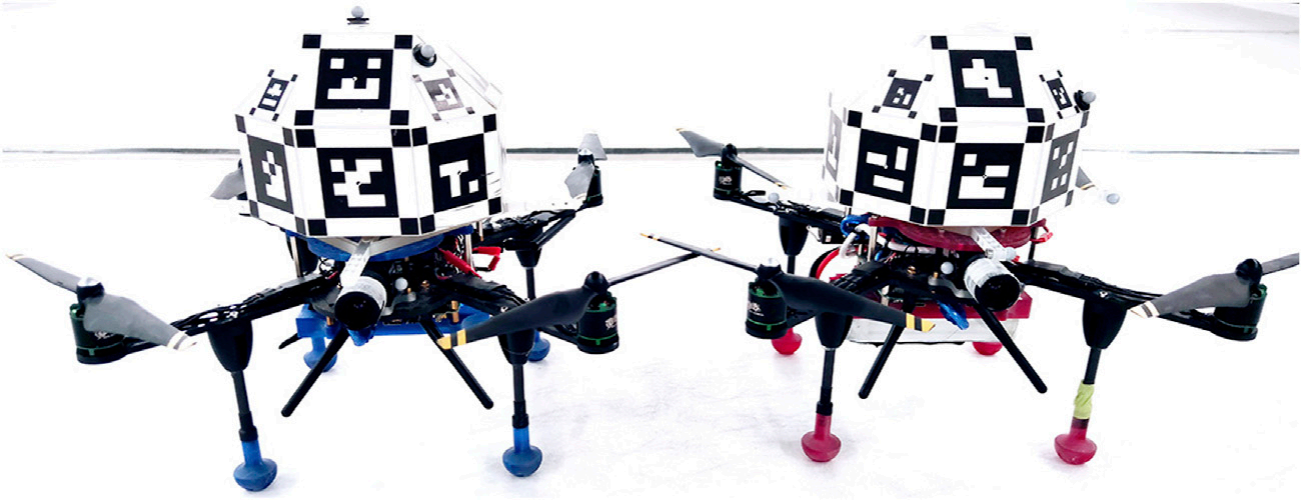
Developed drone-configuration for pose estimation evaluation.

An indicative setup of two drones bearing imaging modalities is showcased in Figure 1 for evaluating the proposed technique in an experimental real-world leader-follower scenario.

In this work, we assume the existence of multiple UAV agents, each bearing an imaging modality without zooming capabilities, while their cameras are assumed to be mounted at the front of the vehicle. Having already introduced several methods for pose detection in Section 1, this research work focuses and extends the use of the ArUco fiducial markers framework (Romero-Ramirez et al., 2018). The identification process consists of a filtering process with local adaptive thresholding for edge extraction. This is followed by a contour extraction and a polygonal approximation to identify the rectangular markers. Then a size based elimination and marker code extraction is carried out, to be followed by marker identification and marker pose estimation. When applied to visual object pose tracking, assuming object distances of less or equal to 5m, this framework can provide a robust and accurate result of position and orientation (Xavier et al., 2017).

**FIGURE 2:**
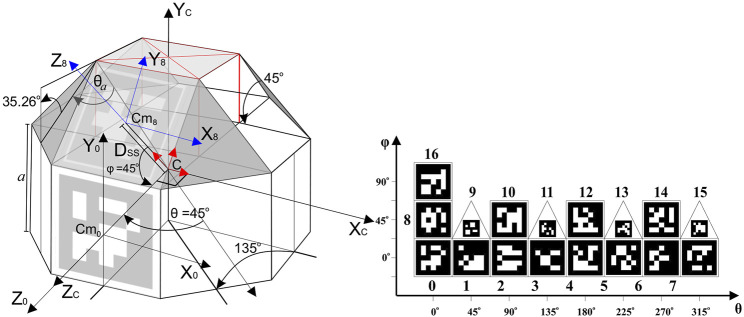
Truncated rhombicuboctahedron geometry and fiducial marker arrangement.

Each UAV is carries the fiducial marker arrangement similar to the one in Tsoukalas et al. (2018). This so called RhombicubOctahedron (RhOct) arrangement is comprised of squares and isosceles triangles as its facets and has an overall weight of 75g. The utilization of this Archimedean-solid, depicted in Figure 2 (left), allows for concurrent observation of these fiducial markers, as shown in Figure 2 (right).

The truncated RhOct comprises of 13 squares and 4 triangle facets and has an area of $A_{r}=13\left(a\times a\right)+4\left(\frac{1}{2}a\times \left(a\sqrt{\frac{5}{4}}\right)\right)\;$ , where *a* is the square facet’s edge length. The planar-rollover of the 17 facets with their associated markers are presented in Figure 2 (right). Marker 0 corresponds to the one that has axis *Z*_*c*_ = *Z*_0_ passing thru its center as shown in Figure 2, and the angles *θ*_*i*_ and *ϕ*_*i*_ for each marker correspond to the horizontal and vertical axes, respectively.

**FIGURE 3:**
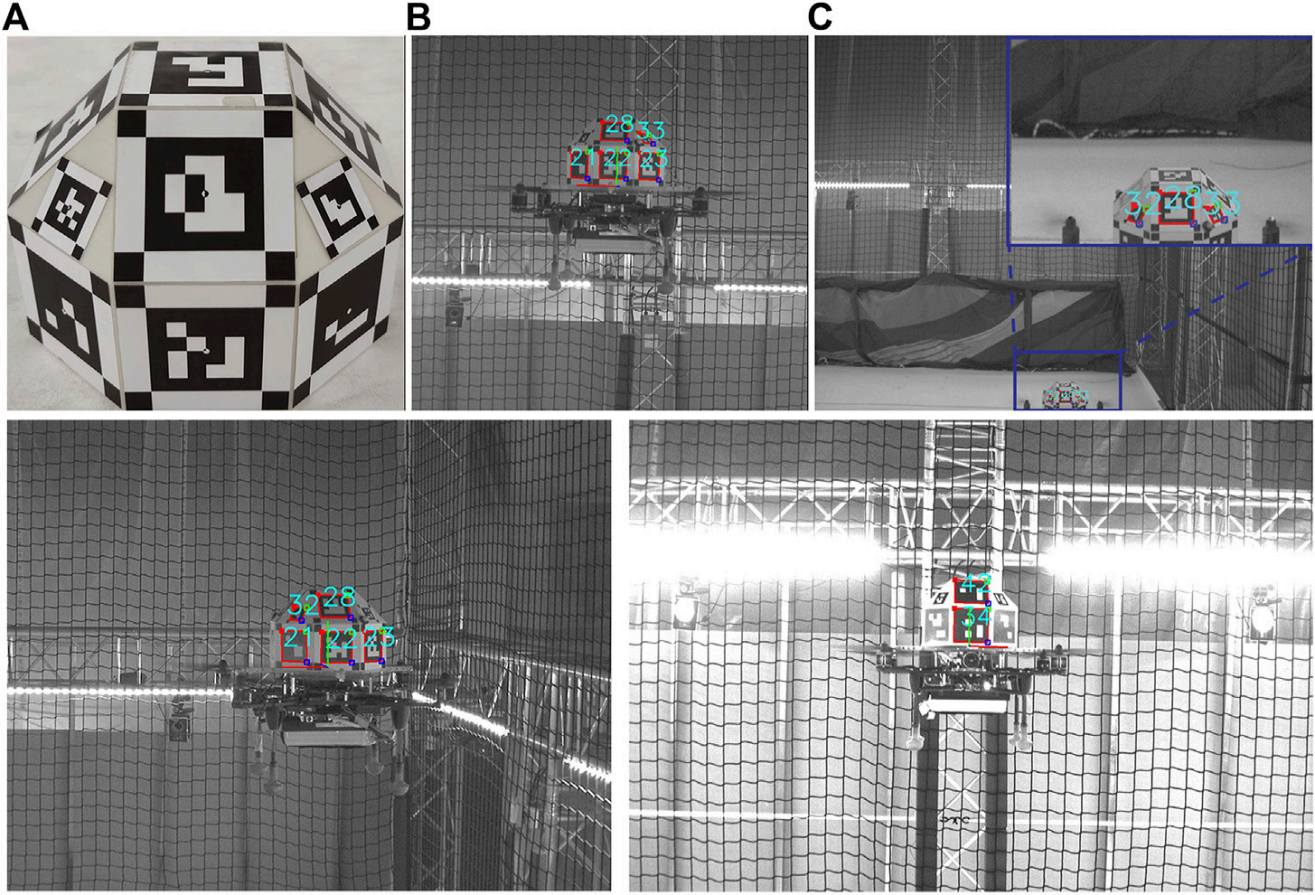
RhOct configuration **(A)**, Detected marker **(B)**, Occlusion case **(C)** and detection under various lighting conditions **(D)**.

This configuration is expected to improve the pose estimation accuracy when more than one marker is detected. Additionally, it ensures robustness in occlusion and lighting conditions, as the system can identify the pose given only a subset of the markers. Such scenarios are depicted in Figure 3. In Figure 3C, the RhOct is partially occluded from the view, however its upper markers are still detected, allowing for the pose estimation process to conclude. Similarly, in Figure 3D two frames are shown with significant varying lighting conditions compared, and yet the RhOct’s pose can be extracted.

The algorithm for pose estimation is initialized by generating each fixed marker’s configuration on the RhOct. The marker identifying number and its corner geometry on the solid are obtained from a configuration file. The marker’s corner geometry, in this case refers, to the coordinates of its four corners with respect to the axis system *X*_*c*_, *Y*_*c*_, *Z*_*c*_ of Figure 2. Subsequently the online pose extraction is carried out for every new image acquisition using the following steps: 1) Detect fiducial (ArUco) markers in the image, 2) For each marker discovered extract the pixel coordinates of each corner, 3) Feed all pixel coordinates to a Perspective *N*–point solver of OpenCV library (Bradski, 2000) for extracting the RhOct’s pose with respect to the camera axis system. The algorithm encountered in the EPnP-formulation (Lepetit et al., 2009) was used to solve Perspective *N*-points (PnP) problem (Quan and Lan, 1999), due to its recursive nature which starts from the previously encountered solution. This algorithm is a significant enhancement to (Tsoukalas et al., 2018), where the center of the solid was extracted from each individual marker and then averaged, leading to ambiguity in individual marker pose extraction (Oberkampf et al., 1996). To address this, the algorithm simultaneously passes all marker corners (in pixels) to the solvePnP function, which in turn calculates the global optimal pose satisfying the initial fixed geometry of the RhOct.

Additional improvements include changes in the marker arrangement for more accuracy and improved detection frequency using a Region-of-Interest (RoI) approach. The adopted dictionary was “ARUCO_MIP_16h3” since this provides 4 × 4 markers which are easier to detect at larger distance than the default ArUco 6 × 6 ones. The markers at the square (triangular) facets were set to 48.75 (22.5)mm to accommodate space for the detection method. This method improved the accuracy, reduced jittering due to the sub-pixel corner refinement.

**FIGURE 4:**
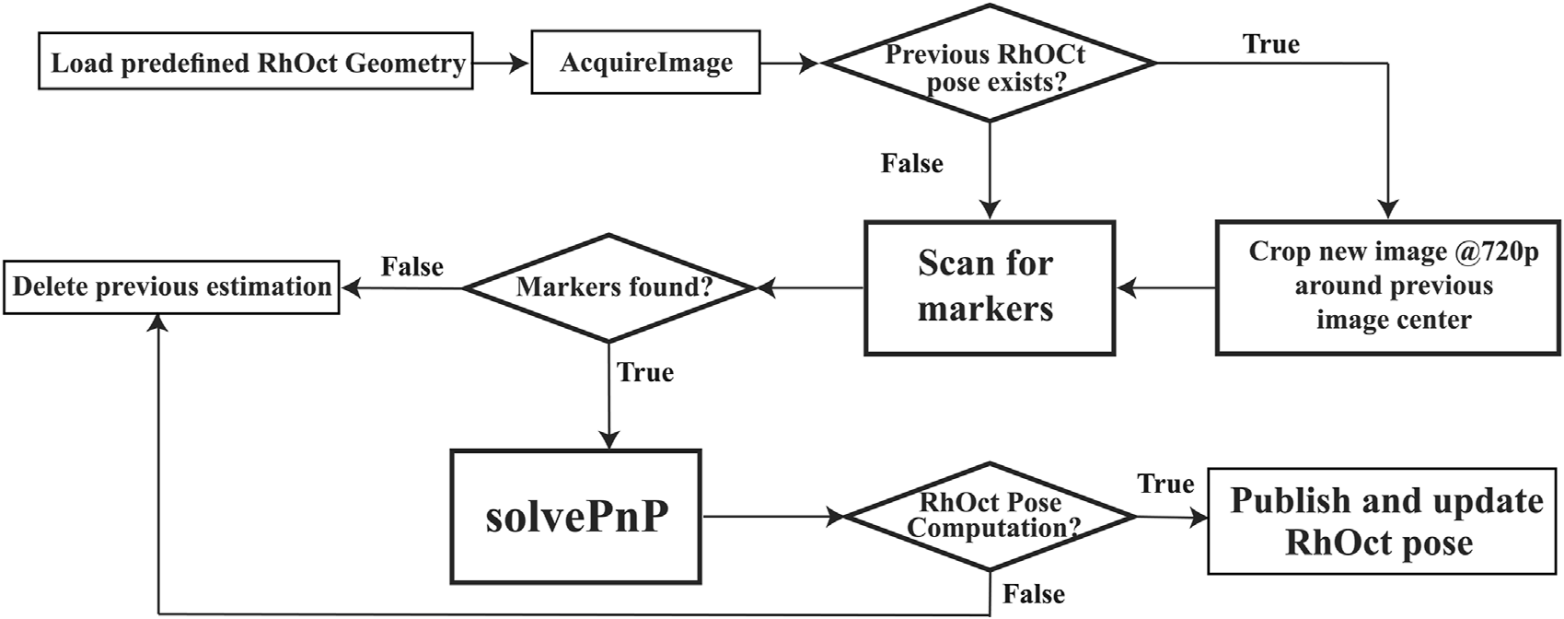
Flowchart of RhOct pose estimation algorithm.

The adopted recursive RoI implementation assumes that a pose solution from the previous iteration exists, and the subsequent image is only cropped at a 1280 × 720 size around the center pixel of that solution and then passed to the pose detection algorithm. This implementation can handle 30 FpS in an i7-CPU implementation and the resulting algorithm is summarized in Figure 4.

## 3 Linearized UAV-Dynamics

Owing to inherent delays in the relative pose computation of the leader demands the inference of the maximum allowable lag, so that proper control parameters can be applied to the follower in order to efficiently maintain the leader within its Field-of-View (FoV).

The dynamic model of a quadrotor with mass *m* forms a set of nonlinear ODEs Sabatino, 2015. Let $o_{p}={\left[x,y,z,\phi ,\theta ,\psi \right]}^{T}\in R^{6}$ the vector containing position and the Euler angle orientation of the quadrotor in earth frame. Let its derivative be ${\dot{o}}_{p}={\left[u,\upsilon ,\omega ,p,q,r\right]}^{T}\in R^{6}$ containing the linear and angular velocities with respect to body frame. Let the UAV’s inertia matrix with respect to its center of gravity be diagonal $I=\left[\begin{array}{ccc} I_{x} & 0 & 0\\ 0 & I_{y} & 0\\ 0 & 0 & I_{z} \end{array}\right]\in R^{3x3}.$ The input forces and torques to the system are assumed proportional to the square rotational speed of the rotors as in Eq. 1, where *l* is the distance of any rotor from the drone center, *b* the thrust factor and *d* the drag factor.

$$\begin{array}{ll} u & ={\left[u_{1},\ldots ,u_{4}\right]}^{T}={\left[f_{t},\tau_{x},\tau_{y},\tau_{z}\right]}^{T}\in R^{4},\;where\\ f_{t} & =b\left(\Omega_{1}^{2}+\Omega_{2}^{2}+\Omega_{3}^{2}+\Omega_{4}^{2}\right)\\ \tau_{x} & =bl\left(\Omega_{3}^{2}-\Omega_{1}^{2}\right)\\ \tau_{y} & =bl\left(\Omega_{4}^{2}-\Omega_{2}^{2}\right)\\ \tau_{z} & =d\left(\Omega_{2}^{2}+\Omega_{4}^{2}-\Omega_{1}^{2}-\Omega_{3}^{2}\right). \end{array}$$ (1)

Let the gyroscopic moments and any ground effect phenomena be absent, then the dynamic model of the quadrotor can be described as in

$$\dot{x}=f\left(x\right)+\overset{4}{\underset{i=1}{\sum }}g_{i}\left(x\right)u_{i},\;where$$ (2)

$x={\left[x,y,z,\phi ,\theta ,\psi ,\dot{x},\dot{y},\dot{z},p,q,r\right]}^{T}\in R^{12}$ , and $c\left(\cdot \right)\overset{△}{=}\cos\left(\cdot \right),\;s\left(\cdot \right)\overset{△}{=}\sin\left(\cdot \right)$ , and $t\left(\cdot \right)\overset{△}{=}\tan\left(\cdot \right)$ .

$$f\left(x\right)=\left[\begin{array}{c} \dot{x}\\ \dot{y}\\ \dot{z}\\ q\frac{s\left(\phi \right)}{c\left(\theta \right)}+r\frac{c\left(\phi \right)}{c\left(\theta \right)}\\ q\left[c\left(\phi \right)\right]-r\left[s\left(\phi \right)\right]\\ p+q\left[s\left(\phi \right)t\left(\theta \right)\right]+r\left[c\left(\phi \right)t\left(\theta \right)\right]\\ 0\\ 0\\ g\\ \frac{\left(I_{y}-I_{z}\right)}{I_{x}}qr\\ \frac{\left(I_{z}-I_{x}\right)}{I_{y}}pr\\ \frac{\left(I_{x}-I_{y}\right)}{I_{z}}pq \end{array}\right],\;and$$

$$\begin{array}{ll} g_{i}\left(x\right) & ={\left[0_{1\times 12}\right]}^{T},i=1,\ldots ,4 \end{array}$$ (3)

$$\begin{array}{ll} g_{1,7}^{T} & =-\frac{1}{m}\left[s\left(\phi \right)s\left(\psi \right)+c\left(\phi \right)c\left(\psi \right)s\left(\theta \right)\right],\\ & g_{1,8}^{T}=-\frac{1}{m}\left[c\left(\psi \right)s\left(\phi \right)-c\left(\phi \right)s\left(\psi \right)s\left(\theta \right)\right] \end{array}$$ (4)

$$\begin{array}{ll} g_{1,9}^{T} & =-\frac{1}{m}\left[c\left(\phi \right)c\left(\theta \right)\right],g_{2,10}^{T}=\frac{1}{I_{x}},\;\;\\ & g_{3,11}^{T}=\frac{1}{I_{y}},\;\;g_{4,12}^{T}=\frac{1}{I_{z}}. \end{array}$$ (5)

Linearizing around a hovering condition $x{}^{\circ}={\left[x‐{}^{\circ},y‐{}^{\circ},z‐{}^{\circ},0_{1\times 9}\right]}^{T}$ and $u{}^{\circ}={\left[mg,0,0,0\right]}^{T}$ to account for the weight of the quadrotor, where x = x° + Δx and u = u° + Δu.

$$\begin{array}{cccccc} \Delta \dot{x}=\Delta \dot{x}, & \Delta \dot{y}=\Delta \dot{y}, & \Delta \dot{z}=\Delta \dot{z}, & \Delta \dot{\phi }=\Delta \dot{\phi }, & \Delta \dot{\theta }=\Delta \dot{\theta }, & \Delta \dot{\psi }=\Delta \dot{\psi }\\ \Delta \ddot{x}=-g\theta , & \Delta \ddot{y}=g\phi , & \Delta \ddot{z}=\frac{1}{m}\Delta u_{1}, & \Delta \ddot{\phi }=\frac{1}{I_{x}}\Delta u_{2}, & \Delta \ddot{\theta }=\frac{1}{I_{y}}\Delta u_{3}, & \Delta \ddot{\psi }=\frac{1}{I_{z}}\Delta u_{4}. \end{array}$$

These equations can be written in a compact form where the notation **I**_*N*_ (**0**_*N*_) refers to an identity (zero) *N* × *N* matrix.

$$\begin{array}{ll} \Delta {\dot{x}}_{12\times 1}= & A_{12\times 12}\;\Delta x_{12\times 1}+B_{12\times 4}\;\Delta u_{4\times 1}. \end{array}$$ (6)

$$\begin{array}{ll} = & \left[\begin{array}{cc} 0_{6} & I_{6}\\ \left[\begin{array}{cc} 0_{3} & \left[\begin{array}{ccc} 0 & -g & 0\\ g & 0 & 0\\ 0 & 0 & 0 \end{array}\right]\\ 0_{3} & 0_{3} \end{array}\right] & 0_{6} \end{array}\right]\Delta x+\\ & \left[\begin{array}{c} 0_{8\times 4}\\ \left[\begin{array}{cccc} \frac{1}{m} & 0 & 0 & 0\\ 0 & \frac{1}{I_{x}} & 0 & 0\\ 0 & 0 & \frac{1}{I_{y}} & 0\\ 0 & 0 & 0 & \frac{1}{I_{z}} \end{array}\right] \end{array}\right]\Delta u, \end{array}$$ (7)

## 4 Controller Design and Maximum Allowable Delay for Leader/Follower UAV-Formation

**FIGURE 5:**
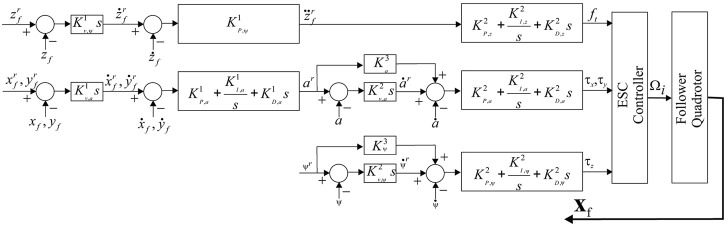
Ardupilot-based controller design for single UAV.

The controllers for the altitude and attitude, as shown in Figure 5 within the Ardupilot framework are adopted in this study. For the *z*-axis a P-differentiator converts vertical position error to vertical velocity; this is subsequently converted to desired vertical acceleration through a cascade P-controller and finally through a PID-controller to motor output. For the *xy*-plane control a gain differentiator coverts the *x* and *y* position error to reference velocity, followed by a velocity PID which converts the velocity error to roll (pitch) when *x* (*y*) is referred. Correspondingly this amounts to *a* = *ϕ* (*θ*) when $x_{f}^{r}\;\left(y_{f}^{r}\right)$ is used. This reference roll (pitch) is sent to the attitude controller which is a second velocity P-differentiator along with a feedforward term which generates the desired roll (pitch) rate. A second cascade PID-controller is used to generate the necessary torques along the *x* (*y*) axes. The yaw-controller is similar to the attitude component of the roll and pitch components, as shown in Figure 5.

The four inputs *f*_*t*_, *τ*_*x*_, *τ*_*y*_ and *τ*_*z*_ are then used in Eq. 1 to compute the Ω_*i*_, *i* = 1, … , 4 which are provided in the ESCs to power up the brushless motors of the quadrotor.

The resulting closed loop stable system takes the torm

$$\Delta \dot{x}=A_{c}\;\Delta x+B_{c}\left[\begin{array}{c} z_{f}^{r}\\ x_{f}^{r}\\ y_{f}^{r}\\ \psi_{f}^{r} \end{array}\right],$$ (8)

where the subscript “*f*” indicates the follower quadrotor.

In a leader/follower configuration the deployment strategy and, thus, reference paths dynamically change upon changes to the position of the leader. Having obtain a dynamic model for a single drone, namely the follower’s dynamic behaviour, the potential coupling between a dynamically moving leader and the follower’s reference path strategy allows for the generation of a single linear model for the multi-agent UAV configuration, that is used for assessing the system stability. It should be noticed that for a single leader/follower UAV configuration this coupling is not necessary.

In a leader/follower configuration we use the subsript “*l*” to distinguish the leader from the follower quadrotor. The leader quadrotor performs a motion while the follower attempts to follow the leader’s motion at a certain distance in each axis, or *x*_*l*_−*x*_*f*_ = *c*_*x*_, *y*_*l*_−*y*_*f*_ = *c*_*y*_ and *z*_*l*_−*z*_*f*_ = *c*_*z*_ where *c*_*x*_, *c*_*y*_ and *c*_*z*_ are constants and $c^{2}=c_{x}^{2}+c_{y}^{2}+c_{z}^{2}$ . It should be noted that the presented controllers are continuous in nature, whereas in reality they are implemented with a 400Hz rate on a Pixhawk autopilot. This generates minor inherent delay in the system response, which in most cases can be neglected. However, in a real world scenario, other inherent time delays in pose estimation can affect the follower’s tracking performance of the leader. These delays can be attributed to sensing modality refresh rates and communication (if needed) of the relative poses within collaborating aerial agents.

In this work the use of an imaging modality for computing the relative pose between the leader and follower, means that the information does not need to be communicated between agents. Still, the refresh rate for the controller output cannot exceed the refresh rate of the imaging modality. In our case this is 30Hz, however for high resolution cameras of 12 mega-pixel it can be as low as 5Hz, which might affect tracking performance in the case of aggressive attitude motions from a leader. This scenario is of similar nature with communicating GPS sensed positioning between agents in outdoor environments for relative positioning. Since these sensors are bounded to a 4Hz frequency, the position of a leader will be communicated to a follower at 250msec for processing the calculations. Coupled to the data RF-transmission delay this interval can easily increase up to 350msec (excluding any lost packets). The follower will then need to compute $x_{f}^{r}:x_{l}-x_{f}≃c_{x}\;\left(y_{f}^{r}:y_{l}-y_{f}≃c_{y}\right)\;\left[z_{f}^{r}:z_{l}-z_{f}≃c_{z}\right]$ and direct it to its controller shown in Figure 5.

Hence there is an inherent varying delay in the feedback path since the vector ${\left[x_{f}^{r}\left(t\right),y_{f}^{r}\left(t\right),z_{f}^{r}\left(t\right)\right]}^{T}$ is computed after *h* seconds, where *h* ∈ [0, 350msec]. Subsequently, there exists a motivation to assess such delays when operating multi-agent systems. We should note that despite *h*(*t*) is time varying, we can use polling to constrain it at a sampling period (in the experimental section $h\in \left\{0.25,\;1,\;1.25\right\}$ seconds. The controller employed by the follower is a simple MIMO *P*-controller, and we should note that the yaw is regulated to zero and there is no need to infer the leader’s relative yaw; this is due to the used RhOct that can be viewed from any yaw angle of the leader. The employed controller is

$$\left[\begin{array}{c} z_{f}^{r}\left(t\right)\\ x_{f}^{r}\left(t\right)\\ y_{f}^{r}\left(t\right)\\ \psi_{f}^{r}\left(t\right) \end{array}\right]=\left[\begin{array}{cccc} K_{z} & 0 & 0 & 0\\ 0 & K_{x} & 0 & 0\\ 0 & 0 & K_{y} & 0\\ 0 & 0 & 0 & 0 \end{array}\right]\left[\begin{array}{c} z_{l}\left(t-h\right)-z_{f}\left(t-h\right)\\ x_{l}\left(t-h\right)-x_{f}\left(t-h\right)\\ y_{l}\left(t-h\right)-y_{f}\left(t-h\right)\\ \psi_{f}\left(t-h\right) \end{array}\right]+\left[\begin{array}{c} c_{z}\\ c_{x}\\ c_{y}\\ 0 \end{array}\right].$$ (9)

The follower quadrotor’s time-delayed dynamics is expressed as

$$\Delta {\dot{x}}_{f}=A_{f}\;\Delta x_{f}\left(t\right)+A_{1,f}\;\Delta x_{f}\left(t-h\right)+B_{f}\left[\begin{array}{c} c_{z}\\ c_{x}\\ c_{y}\\ 0 \end{array}\right].$$ (10)

This system is asymptotically stable for any constant delay *h*: 0 < *h* < *h* ^max^ of there exist matrices *p* > 0, *Q* > 0 and *Z* > 0 such that (Gouaisbaut and Peaucelle, 2006; Xu and Lam, 2008)

$$\left[\begin{array}{ccc} PA_{f}+A_{f}^{T}P+Q-Z & PA_{1,f}+Z & h^{\max}A_{f}^{T}Z\\ A_{1,f}^{T}P+Z & -Q-Z & h^{\max}A_{1,f}^{T}Z\\ h^{\max}ZA_{f} & h^{\max}ZA_{1,f} & -Z \end{array}\right]<0$$ (11)

An exhaustive search is needed to compute the maximum delay that is allowed under the time-delayed leader/follower configuration. It should be noted: 1) that the provided results are sufficient and higher values of *h* ^max^ can still provide a stable system, and 2) the provided analysis is valid for near hovering conditions by the follower forcing a smooth trajectory [*s*(*ϕ*) ≃ *ϕ* and *s*(*θ*) ≃*θ*, or *ϕ*, *θ* ≤ 10°]. Furthermore the inherent assumption is that the leader remains within the FoV of the follower’s camera and the marker can be viewed with clarity.

## 5 Experimental Studies

In the following experimental study, the efficiency and accuracy of the suggested scheme is evaluated using two identical drones in a leader/follower formation.

### 5.1 Experimental Setup

**FIGURE 6:**
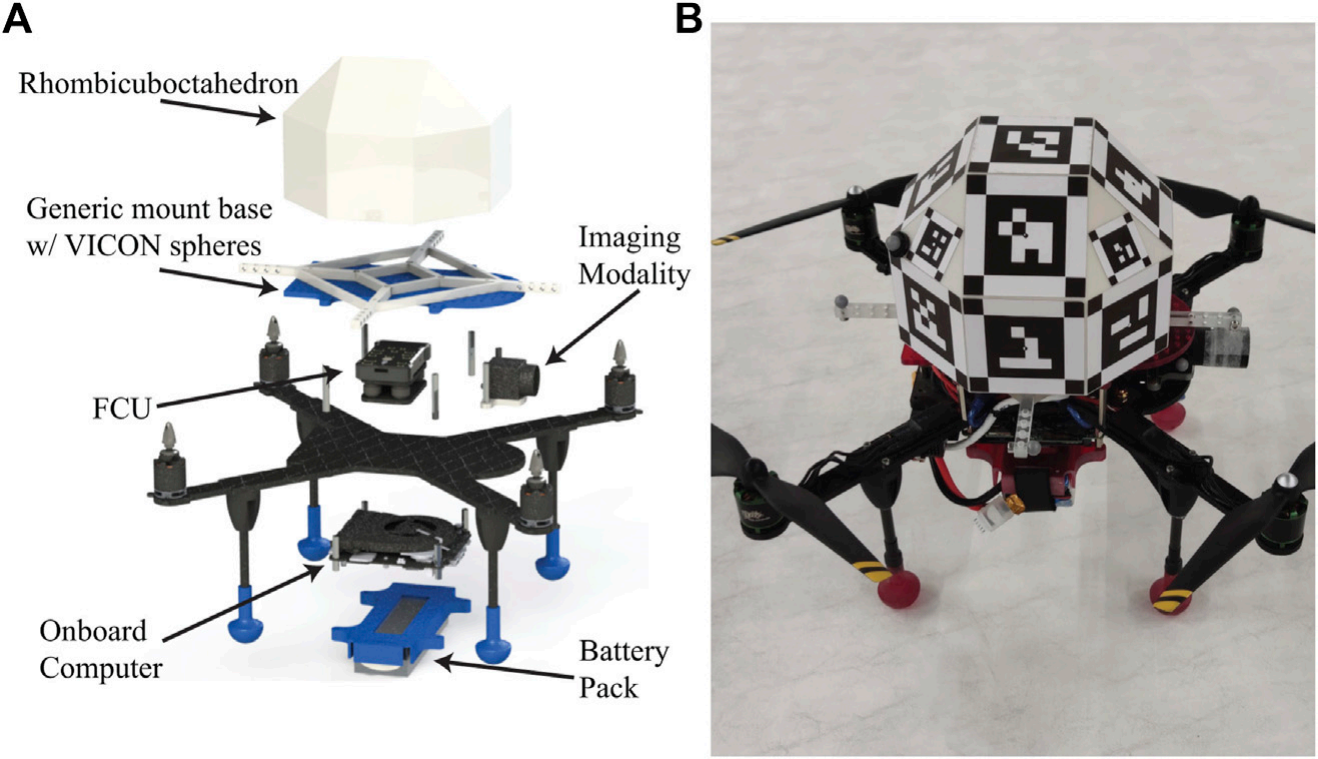
Developed UAV-rendering **(A)** and physical prototype **(B)**.

The utilized quadrotors bear an imaging modality and a unique RhOct arrangement described in Section 2. Moreover, an Intel NUC i7 onboard computer and a PixHawk flight controller with the open-source ArduPilot (ArduPilot, 2021) framework are fitted for processing power and carrying out the low level flight actions respectively. The total weight of each UAV is 2.2kg with an allowable flight time of 11min using a 4-Cell LiPo battery. A FLIR BlackFlyS camera is used for image acquisition with a 2048 × 1534pixel resolution; the achievable acquisition rate using the onboard computer reached 37 FpS. The camera was calibrated using the ArUco calibration board for a fixed focus at 2.75m distance. 8-bit grayscale images were acquired to conform with the ArUco framework image processing methods, while the shutter speed was kept at 5nsec for minimizing blurriness. The utilized algorithms rely on the ROS (Quigley et al., 2009) framework and can be accessed from the following Github repository. A rendered version and the actual prototype are depicted in Figure 6 respectively. A motion capture system, comprising of 24 high resolution VICON cameras (Vicon motion systems, 2021) operating at 120 Hz was utilized for validating drones’ pose with 0.5mm and 0.5° accuracy.

### 5.2 Relative Pose Measurement With Stationary UAVs

**FIGURE 7:**
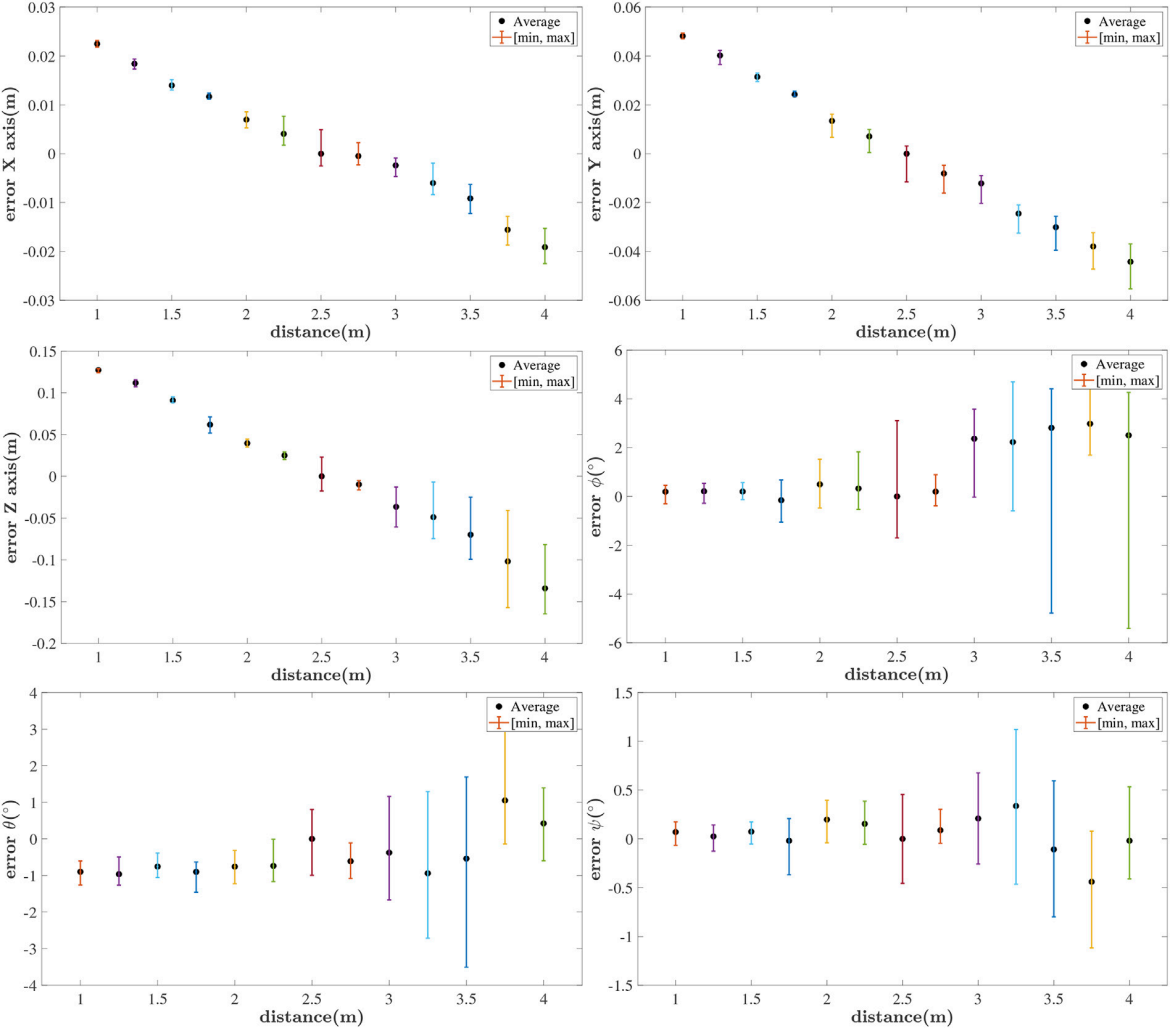
Attainable pose measurement error for stationary UAVs at different distances $\left|x_{l}-x_{f}\right|$ .

This section investigates the maximum allowable detection distance for the RhOct configuration and the relative pose error as a function of the leader/follower distance. Both stationary UAVs were set so as to increase the distance between them |*x*_*l*_−*x*_*f*_| ∈ {1, 1.25, … , 4}m. At each distance we perform pose estimation of the leader’s RhOct, using the follower’s imaging modality for a duration of 120s. In Figure 7 the average and peak values of the pose estimation errors are depicted.

All translational and rotational errors seem to drift away as the distance varies away from the optimal focal length distance of 2.75m. The algorithm operates extremely well at distances up to 4 m with a typical translational (rotational) error (3 × 4 × 15)cm (3° × 0.5° × 0.1°).

### 5.3 Relative Pose Measurement With Hovering UAVs

**FIGURE 8:**
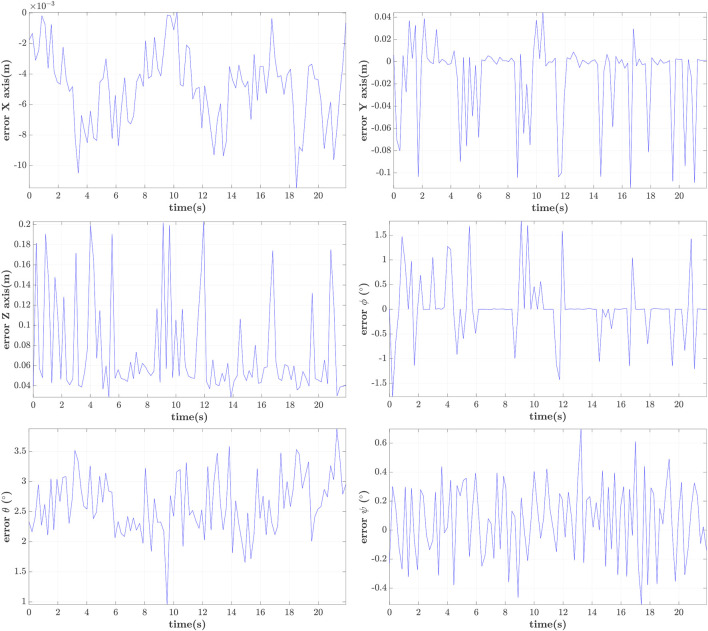
Visual vs. Motion Capture System induced pose measurement error.

In order to account for the induced UAV-vibrations in measuring their pose, two drones in a hovering condition facing each other are used in this study. The relative error measured by our visual algorithm compared to the one inferred by the motion capture system is shown in Figure 8, where it is shown that the mean (worst) translational error was 4.9 × 13.8 × 73.9mm (11.5 × 113.8 × 203.7mm) for the *XYZ* axes respectively. These small errors are mainly attributed to the ArUco corner refinement algorithm and are consistent with the large deviations reported in the *Z*-axis (Ortiz-Fernandez et al., 2021).

**FIGURE 9:**
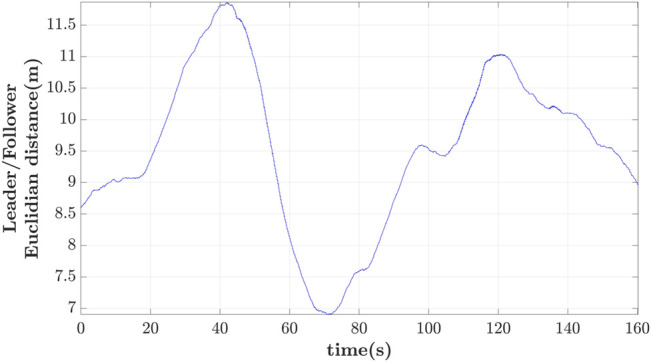
GPS-altitude relative error for stationary UAVs.

For comparison purposes the relative position relying on raw GPS measurements for two stationary drones is shown in Figure 9. Thee drones are placed at 2.75m relative distance and their GPS receivers recorded their translational coordinates for 160s from six available satellites. The typical error was two orders of magnitude larger compared to the one from the visual system and is in agreement with the one reported in the literature; the rotational error from these measurements was constrained to 5° × 5° × 6°.

### 5.4 Leader-Follower Scenario Evaluation

In this scenario the leader is commanded to execute two consecutive skewed rectangular paths with predetermined waypoints, while these two rectangles are distanced from each other at 1m. The follower tracks the leader at a distance with parameters (*c*_*x*_, *c*_*y*_, *c*_*z*_) = (0, 2, 0)m using the controller from Eq. 9. The relative leader/follower distance is estimated through the leader’s detected RhOct. When the leader cannot be detected (i.e., being outside of the follower’s FoV or due to occlusion from obstacles) the follower remains in a hovering mode till the subsequent’s leader’s detection. The theoretical maximum allowable latency *h* ^max^ was 1.9s and for emulation purposes such a delay is induced into calculating the follower’s reference altitude. Similarly to the previous cases, the positions of the two UAVs are compared using the VICON system for delays of 0.25, 1 and 1.25s.

**FIGURE 10:**
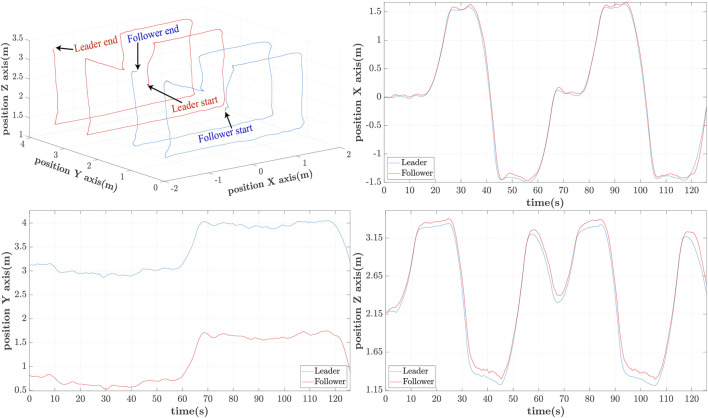
3D Leader/Follower trajectories with *h* = 0.25s delay.

**FIGURE 11:**
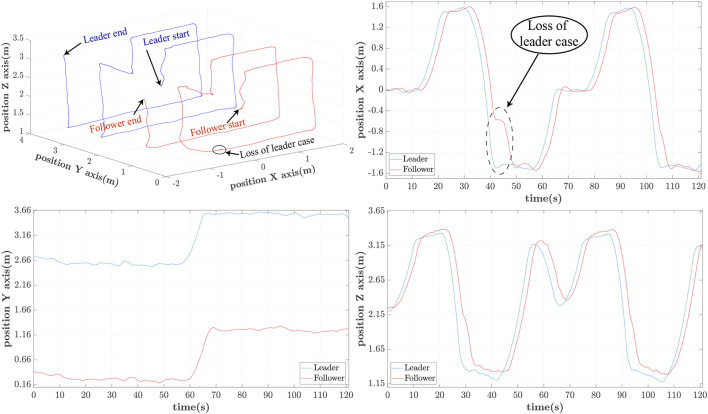
3D Leader/Follower trajectories with *h* = 1s delay.

**FIGURE 12:**
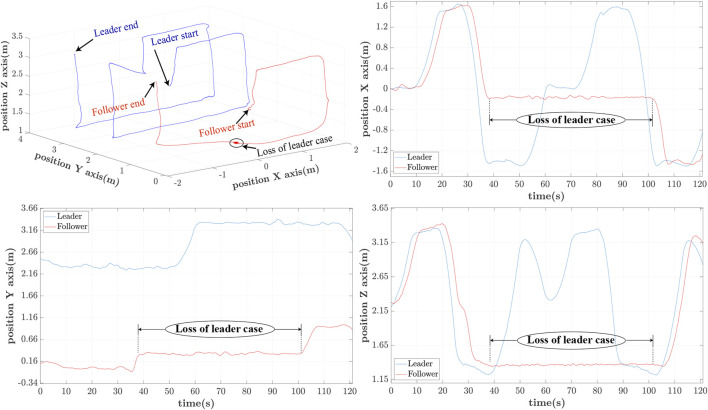
3D Leader/Follower trajectories with *h* = 1.25s delay.

In Figures 10–12 the 3D-path of the leader and follower is shown in the top-left portion, while their *X*, *Y* and *Z* trajectories are shown in the top-right, bottom-left and bottom-right parts respectively. These are expressed in the ENU frame configuration (*X*-forward, and *Z*-upward). For delays of *h* = 0.25s, shown in Figure 10, the follower closely follows the path of the leader with minimal lag. The leader completes its trajectory at 125s with a small translational velocity, so that it remains as much as possible within the follower’s FoV.

Similarly for *h* = 1s the path patterns are shown in Figure 11. Again we notice that the follower can accurately follow the path of the leader with an approximate phase lag of 2s. This delay is attributed to the UAV’s controller and the imposed *h* = 1s delay of applying the estimated reference path. It is also worth noticing a partial loss of the leader at 42s for a brief period of 3s due to the leader’s disappearance out of the follower’s-camera FoV.

**FIGURE 13:**
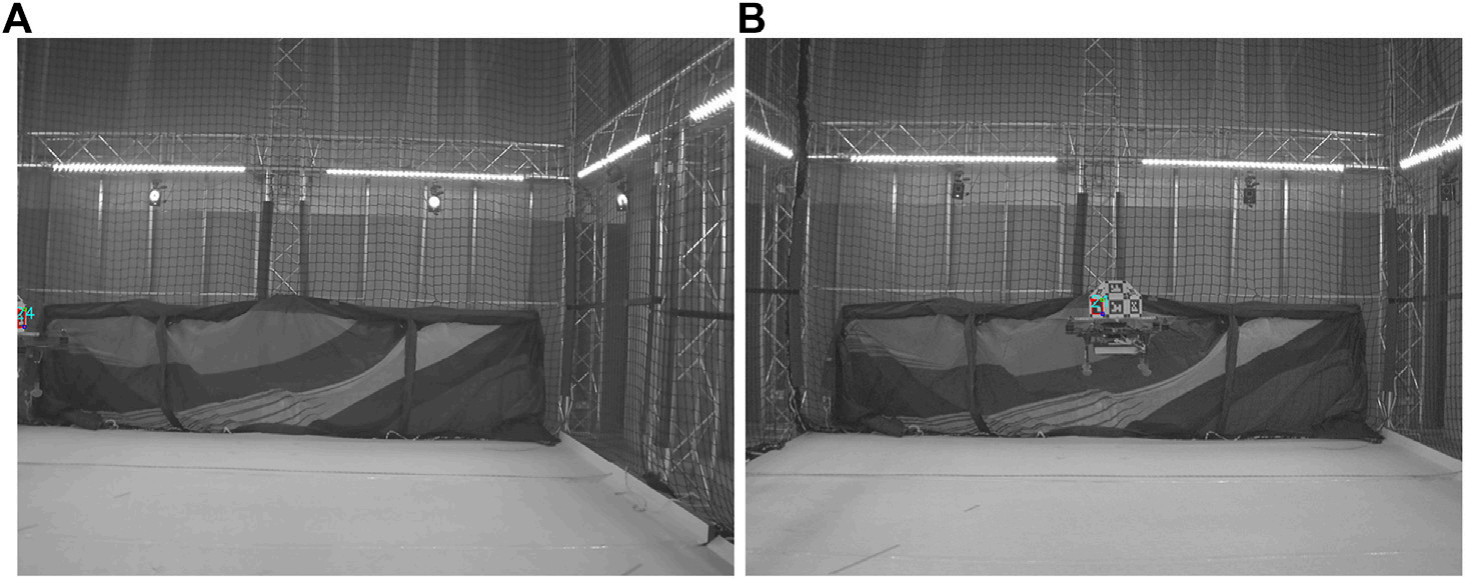
Follower’s camera FoV upon loss **(A)** and reappearance **(B)** of leader.

The last iteration of *h* = 1.25s delay is included to showcase the behaviour of the leader/follower configuration upon loss of leader’s reference path. This is happening owing to the large delay, which leads to the leader’s moving out of the follower’s FoV. In this case, the follower switches to a hovering case per the adopted strategy, only to re-initiate the controller when the leader reappears within FoV at *t* ≃101s. This case appears in Figure 12, whereas the moments of losing and regaining view of the leader’s RhOct are visualized in Figure 13.

**FIGURE 14:**
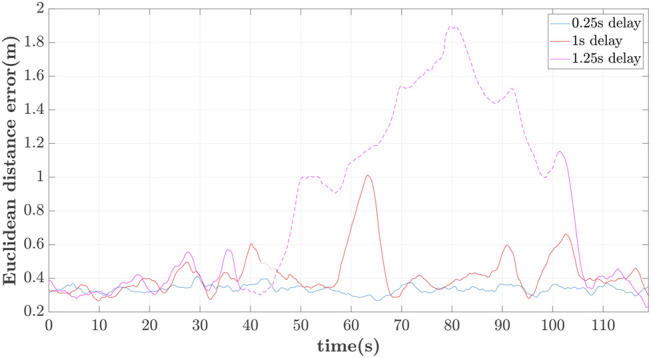
Follower’s Euclidean distance error vs. delay.

The relative Euclidean distance errors between the follower’s reference and actual position are depicted in Figure 14 for different delays. It should be noticed that the error is still computed when the leader is not within the FoV of the follower. As expected, there is significant drift of the follower’s reference path as the delay increases. The mean value of the error is: 1) 0.33m for 0.25s delay, 2) 0.41m for 1s, and 3) 0.81m for 1.25s delay. The loss of the leader is highlighted with the dashed line-segment of the error for the case of 1 and 1.25s delay.

### 5.5 Extension to a Multiple-Agent Scenario

**FIGURE 15:**
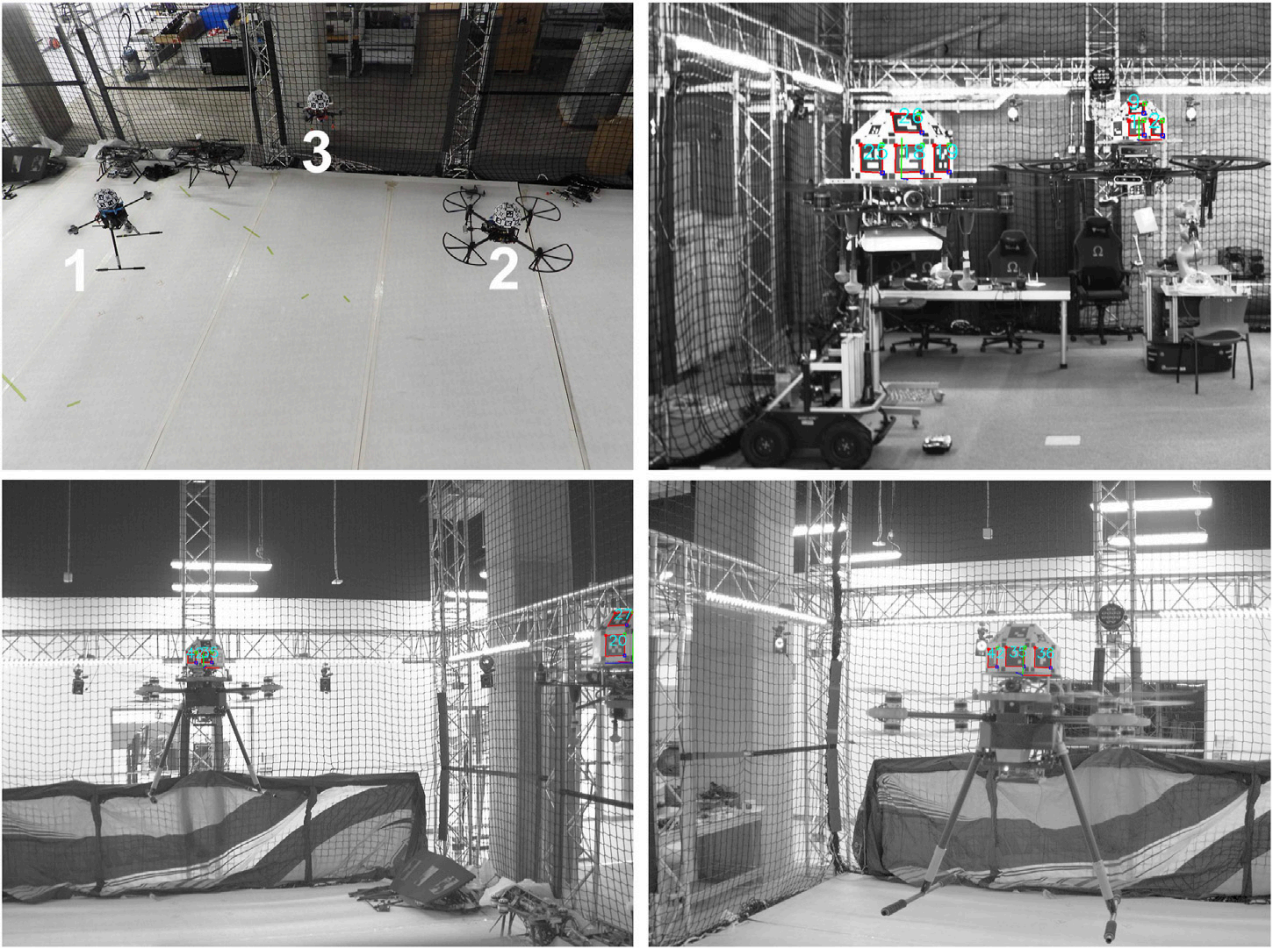
Swarm flying formation and detection view per agent.

The aforementioned scheme can be extended to multi-agent relative localization in a swarm deployment experimentation. Such a case is shown in Figure 15, where three heterogeneous UAVs “1 thru 3” are fitted with a RhOct shown in the top-left portion while the pose detection algorithm is extended to multiple agents within the FoV of each drone. In this experiment drone 1 can view 2 and 3 (top-right), drone 2 can view 1 and partially 3 (bottom-left) while drone three can only observe 1 (bottom-right). A directed observation visual observation graph can be generated in this case having selected one of these as the anchor of the graph, In a leader/several followers scenario in this case, drone “1” is the leader being observable by both “2” and “3”. The only limiting factor in this case is the number of available dictionary of markers, where with a 4 × 4 fiducial dictionary, 1,000 markers can be generated which can be mounted in $58=\left\lfloor \frac{1000}{17}\right\rfloor$ drones. In this case, the occlusion of some markers from the FoV of each agent necessitates the computation of a proper control strategy (Das et al., 2002; Mariottini et al., 2005).

## 6 Conclusions

In experimental leader/follower UAV-configurations, there is need to transmit the relative pose between these UAVs. Rather than relying on the individual FCUs and an RF-transmission scheme, fiducial markers are attached in each UAV. Using an onboard camera, these markers are accurately detected and the relative pose can be inferred within 30msec, as long as the leader is within the follower’s FoV and at a distance up to 4m. The maximum theoretical latency time is compared to the experimental one, while the FoV can be extended: 1) to cover the complete space if spherical cameras are used (Holter et al., 2021), and 2) focus in multiple agents.

## Data Availability

The raw data supporting the conclusion of this article will be made available by the authors, without undue reservation.

## Abbreviations

ENU, East-North-Up; FoV, Field-Of-View; FCU, Flight Control Unit; GNSS, Global Navigation Satellite System; GPS, Global Positioning System; NWU, North-West-Up; PnP, Perspective N-points; ROS, Robot Operating System; RTK, Real-time kinematic; SLAM, Simultaneous Localization and Mapping; UAV, Unmanned Aerial Vehicle; VOT, Visual Object Tracking.
